# The Protective Effects of Perch Essence Against Muscle Atrophy in Cancer Cachexia and Cisplatin Treatment

**DOI:** 10.3390/cimb47030152

**Published:** 2025-02-26

**Authors:** Shu-Lan Yeh, Pei-Yin Chen, Jiunn-Wang Liao, Ruo-Li Huang, Shu-Han Yu, Ling-Ni Chen, Mao-Hsiang Lee, Li-Wen Chen, Haw-Wen Chen, Ya-Chen Yang, Yu-Ling Wu, Kai-Li Liu

**Affiliations:** 1 Department of Nutrition, Chung Shan Medical University, Taichung 40203, Taiwan; suzyyeh@csmu.edu.tw (S.-L.Y.); ruoly.huang@smu.edu.tw (R.-L.H.); yuanniehanhan@gmail.com (S.-H.Y.); 2 Department of Nutrition, Chung Shan Medical University Hospital, Taichung 40203, Taiwan; 3 Department of Senior Citizen Welfare and Long-Term Care Business (Master Program), Hungkuang University, Taichung 433304, Taiwan; peiyinchen@hk.edu.tw; 4 Graduate Institute of Veterinary Pathology, College of Veterinary Medicine, National Chung Hsing University, Taichung 402202, Taiwan; jwliao@dragon.nchu.edu.tw; 5 Anyong Biotechnology Inc., Kaohsiung 827012, Taiwan; celia.chen@topco-global.com (L.-N.C.); sam.lee@topco-global.com (M.-H.L.); 6 Division of Nutrition Therapy, Jen-Ai Hospital, Taichung 412224, Taiwan; jen.6810@mail.jah.org.tw; 7 Department of Nutrition, China Medical University, Taichung 40402, Taiwan; chenhw@mail.cmu.edu.tw; 8 Department of Health and Nutrition Biotechnology, Asia University, Taichung 413, Taiwan; yachenyang@asia.edu.tw; 9 Cardiovascular and Mitochondrial Related Disease Research Center, Hualien Tzu Chi Hospital, Buddhist Tzu Chi Medical Foundation, Hualien 970, Taiwan

**Keywords:** perch essence, cancer cachexia, cisplatin, muscle atrophy

## Abstract

Muscle atrophy, through several pathways including increased protein catabolism, leads to adverse effects in cachexia induced by cancer and chemotherapy. Perch essence (PE) is a perch extract rich in branched-chain amino acids and peptides. The present study initially investigated the effects of PE supplementation on muscle atrophy in a mouse model of cancer cachexia induced by C26 cancer cells and compared these effects with those of tryptone. Compared with the tumor-only group, we found that PE supplementation significantly improved body weight, muscle mass, maximum limb grip strength (MLGS), and myosin heavy chain expression in the muscles of tumor-bearing mice. PE also significantly inhibited the expression of factors related to protein degradation, oxidative stress, and inflammation, while enhancing the expression of antioxidant enzymes in tumor-bearing mice. These effects of PE were associated with an increased expression of phosphorylated Akt and forkhead box protein O1, along with a reduced expression of phosphorylated nuclear factor-κB p65 in the muscles of tumor-bearing mice. Furthermore, PE similarly increased MLGS and attenuated muscle atrophy in mice exposed to cisplatin by inhibiting protein degradation. All the therapeutic effects of PE supplementation mentioned above were generally greater than those of tryptone supplementation. These results suggest the potential of PE in protecting against muscle atrophy induced by tumors or chemotherapy.

## 1. Introduction

Between 50 and 80% of cancer patients suffer from cancer cachexia, and more than 30% of patient deaths are caused by the aftermath of this wasting syndrome, which is associated with decreased physical functions and tolerance for cancer treatments [1]. Chemotherapy is one of the first lines of cancer treatment, but data have shown that cisplatin and some other chemotherapeutic agents can cause or worsen cachexia [2]. Body weight and muscle mass loss have also been observed during chemotherapy, which may involve alterations in nutritional status and protein metabolism in tissues [2,3]. Cancer cachexia is a complex, multifactorial wasting syndrome characterized by unintentional and pathological weight loss, primarily involving the depletion of skeletal muscle mass [4]. Rather than solely increasing caloric intake, studies have shown that nutritional support with specific compositions may help delay the progression of cancer-related muscle atrophy [5].

Although the upstream factors and mechanisms are complex and not fully understood, current evidence suggests that muscle atrophy in cancer cachexia mainly results from persistently imbalanced protein homeostasis, driven by its catabolic condition [6,7]. Based on accumulating evidence, enhanced muscle protein degradation through activation of the ubiquitin-dependent proteasome pathway (UPP) and the autophagic–lysosomal pathway (ALP) is primarily responsible for muscle atrophy and dysfunction in cancer cachexia [6,7,8]. Moreover, the expression of markers related to UPP and ALP is also augmented by chemotherapeutic agents, contributing to muscle atrophy during chemotherapy [2,3,9].

Oxidative stress and inflammation, which are closely interconnected in a feedback loop, act synergistically to initiate and progress muscle atrophy in cachexia [10,11]. Data have shown augmented reactive oxygen species (ROS) levels not only in the serum but also in the muscles of individuals with cachexia [10,12]. In this regard, a correlation has been observed between muscle atrophy and increased ROS in tumor-bearing animals [13], likely due to elevated proteolysis resulting from the activation of the nuclear factor-κB (NF-κB) and forkhead box protein O (FOXO) pathways [7,10]. Systemic inflammation, driven by proinflammatory cytokines such as tumor necrosis factor-α (TNF-α), interleukin (IL)-6, and IL-1β, produced by both the tumor and the host, is involved in the pathogenesis of cancer cachexia. Moreover, this inflammation could trigger an increase in the transcriptional activity of NF-κB, creating an autoregulatory feedback loop that further elevates local muscle inflammation [7,11]. Besides being a central transcription factor for the upregulation of proinflammatory cytokine expression [14], the NF-κB pathway is also involved in muscle atrophy, including cancer cachexia, by enhancing muscle RING-finger protein 1 (MuRF1) expression [15]. Increased phosphorylation and DNA binding activity of NF-kB p65, the most functional subunit of the NF-κB family, have been observed in the muscles with cancer cachexia [15,16]. Additionally, increased transcriptional activity of FOXO transcription factors, which have been revealed to regulate muscle atrophy-related gene expression is valuable in muscle atrophy associated with cancer cachexia [17,18]. Notably, the AKT pathway inhibits protein degradation in the development of muscle atrophy by increasing phosphorylation, thereby decreasing FOXO activation [18]. In summary, oxidative stress, inflammation, as well as the downstream AKT/FOXO and NF-κB pathways, are implicated in the upregulation of UPP- and ALP-related protein expression, which plays a critical role in muscle atrophy in cancer cachexia.

Due to their safety and fewer side effects compared to synthetic drugs, several studies have focused on the effects of nutraceutical supplements, such as fish oil, high-quality protein, and branched-chain amino acids (BCAAs), in preventing or delaying muscle atrophy associated with cancer cachexia and chemotherapeutic agents [19,20,21]. In addition to omega-3 polyunsaturated fatty acids, the proteins from fish may also contribute to the health benefits of fish consumption. In healthy rats, a diet with Alaska pollock protein promotes skeletal muscle hypertrophy and protein synthesis by increasing the AKT/mTOR pathway [22]. Recently, supplementation with perch (*Lates calcarifer*) essence (PE), a concentrated fish protein extract obtained through high pressure and high temperature processing, was shown to improve exercise performance, reduce sports fatigue, accelerate fatigue recovery in mice, and enhance skeletal muscle mass and function in humans [23,24]. Moreover, PE treatment decreased C2C12 myotube death induced by dexamethasone [24]. Based on these data, the present study explored the effects and possible mechanisms of PE on muscle atrophy induced by cancer cachexia and cisplatin (a chemotherapeutic agent) in mice.

## 2. Materials and Methods

### 2.1. Materials

The PE powder was sourced from Anyong Biotechnology Inc. (Kaohsiung, Taiwan) and contains approximately 1 g of protein and 80 mg of branched-chain amino acids (BCAAs) per gram of PE powder [25]. According to the human equivalent dose proposed by the U.S. Food and Drug Administration [26], supplementation with 0.024 g of PE powder per mouse is equivalent to human supplementation with a 60 mL pack of PE, containing approximately 4.71 g of freeze-dried extract. The product was prepared by the method described previously and it is also rich in collagen and taurine [24]. RPMI-1640 media and Tri-ReagentTM were obtained from Invitrogen Corporation (Carlsbad, CA, USA). Fetal bovine serum (FBS) and penicillin–streptomycin solution for cell culture were purchased from HyClone (Logan, UT, USA). Antibodies against Anti-Atrogin-1 and MuRF-1 were acquired from ECM Biosciences (Aurora, CO, USA). Antibodies against AKT and phospho-Akt, FoxO1/FoxO3a, and NF-κB-P65 were purchased from Cell signaling Technology (Danvers, MA, USA). Antibodies against glyceraldehyde-3-phosphate dehydrogenase (GAPDH), Heme Oxygenase-1 (HO-1) microtubule-associated protein 1 light chain 3 (LC3) antibody were obtained from Merck Millipore (Burlington, MA, USA). Antibodies against Beclin-1, myosin heavy chain (MyHC), superoxide dismutase 2 (SOD2) were secured from Santa Cruz Biotechnology, Inc. (Dallas, TX, USA), Thermo Fisher Scientific Inc. (Waltham, MA, USA), and GeneTex, Inc. (Hsinchu City, Taiwan), respectively. Reagents for synthesizing complementary DNA and PowerUpTM SYBRTM Green Master Mix were acquired from Promega Corp. (Madison, WI, USA) and Applied Biosystems (Foster City, CA, USA), respectively.

### 2.2. Animal Study

BALB/c mice aged 4 weeks were obtained from the National Laboratory Animal Center (Taipei, Taiwan) and housed in a temperature-controlled room (25 °C) with a relative humidity of 55%, under a 12 h light–dark cycle, with free access to food and water. All animal experiments were conducted with the approval of the Institutional Animal Care and Use Committee of Chung Shan Medical University (IACUC: 2759 and 2761).

#### 2.2.1. Colon-26 Tumor-Bearing Mouse of Cachexia

Mouse colon cancer cell line colon-26 were gifted from Dr. Chih-Hung Guo and grown in RPMI-1640 media with 10% FBS, 1% penicillin/streptomycin, 3.7 g/L sodium bicarbonate, 3.15 g/L HEPES, and 2 mM L-glutamine at 37 °C in a humidified atmosphere of 5% CO_2_.

Mice were randomly divided into four groups based on weight: a control group (C, *n* = 11), Colon-26 tumor-bearing group (T, *n* = 12), Colon-26 tumor-bearing group supplemented with PE (T+PE, *n* = 12), and Colon-26 tumor-bearing group supplemented with tryptone in the same amount protein as PE (T+TR, *n* = 12). On study day 1, C26 tumor cells (1 × 10^6^/150 µL) were implanted subcutaneously in the right flank of tumor-bearing mouse groups. Until sacrifice on day 21, all mice were fed the AIN-93M diet ad libitum. Groups C and T were given regular drinking water and received a daily intragastric gavage with 150 µL of saline. Based on previous studies with some modifications [23,27], the T+PE group was provided with drinking water containing 1.17% (*w*/*v*) PE powder and received a daily gavage of 0.024 g of PE powder in 150 µL, equivalent to human supplementation with three packs of PE. Food and water intake, body weight, and tumor volume were measured every other day.

#### 2.2.2. CDDP-Induced Mouse Muscle Atrophy

Mice were randomly assigned to the following five groups (*n* = 8/group) for 9 weeks: Control, CDDP, CDDP+1XPE (0.024 g/d), CDDP+2XPE (0.048 g/d), and CDDP+tryptone (0.048 g/d). CDDP (dissolved in a 0.9% saline solution) was administered intraperitoneally (i.p) at a dose of 2.5 mg/kg body weight per week [3,9]. PE powder and tryptone were dissolved in 200 μL distilled water and administered daily by oral gavage. The control group served as the vehicle. All animals were allowed free access to the AIN-93M diet. Body weight and food intake of the animals were recorded every 2 days during the experiment. Similarly, based on the aforementioned data, 1X and 2X PE supplementation correspond to human supplementation with 1 and 2 packs of PE, respectively [23,27].

The dosage of tryptone was determined to provide the same amount of protein as PE supplementation in the same experimental animal models.

#### 2.2.3. Tissue and Blood Sample Collection and Processing

After being sacrificed, blood samples were collected and the plasma sample was separated and used for various biochemical analyses. Meanwhile, tissues from the heart, liver, spleen, kidney, testes, epididymal fat, as well as parts of muscles, including triceps, gastrocnemius, soleus, and tibialis anterior, were collected and stored at −80 °C until analysis. The quadriceps muscles were stored in 10% formalin and then fixed and sectioned for Hematoxylin and Eosin (H&E) staining to determine the fiber size.

### 2.3. Forelimb Grip Strength Test

The animals were subjected to the forelimb grip strength test to measure the maximum grip strength (MGS) using a grip strength meter (Ugo Basile, Gemonio, Italy) at the indicated time points. The details of the test were described previously [21]. The mice were allowed to grip the triangular bar with two forelimbs and then the mouse’s tail was gently pulled back. The maximum force was recorded when the mice released the grasp. The tests were performed three times at one min intervals by the same person using a similar and stable force for each mouse to obtain the average value. The maximum force value was used to reflect muscle weakness.

### 2.4. Muscle Fiber Size

The quadriceps of mice were fixed and embedded in a cassette and immersed in formalin before being sliced and followed by H&E staining [21]. Then, quadriceps samples were examined using a Tissue Cytometer (TissueGnostics, Vienna, Australia; magnification, ×200). The cross-sectional areas (CSAs) of myofibers in the rectus femoris region were calculated using TissueFAXS Viewer software 3.5 (TissueGnostics, Vienna, Australia), and a minimum of 8 random images and 200 sets (25 sets/image) of data were acquired per group.

### 2.5. Total Protein Extraction and Western Blotting

Radioimmunoprecipitation assay lysis buffer and a modified Lowry assay were used to prepare total protein extracts and quantify protein concentration from homogenized GA muscle, respectively [28]. Equal amounts of proteins were denatured and separated on SDS-polyacrylamide gels and were then transferred to polyvinylidene difluoride membranes (New Life Science Product, Inc., Boston, MA, USA). The blots were sequentially incubated with primary antibodies, followed by horseradish peroxidase-conjugated secondary antibodies (Bio-Rad, Hercules, CA, USA). Immunoreactive protein bands were developed using an enhanced chemiluminescence kit (Perkin–Elmer Life Sciences, Boston, MA, USA), visualized with a luminescent image analyzer (LAS-1000 Plus, Fuji Photo Film Company, Tokyo, Japan), and quantified using AlphaEase FC software version 4 (Alpha Innotech Corp., San Leandro, CA, USA).

### 2.6. RNA Isolation and Real-Time Reverse-Transcriptase PCR (Real-Time RT-PCR)

Total RNA was extracted from GA muscle by using Tri-Reagent as described by the manufacturer. RNA extracts were suspended in RNase-free water and were frozen at −80 °C until analyzed. cDNA synthesis was made using M-MLV reverse transcriptase. Subsequently, cDNA was amplified using PowerUp^TM^ SYBR^TM^ Green Master Mix and specific primers, and reactions were quantified on the StepOne System (Applied Biosystems, Norwalk, CT, USA). Primers and probes were obtained from Applied Biosystems, m-MyHC2B, sense: AGACTTCAAGCAGAG ATACA, anti-sense: AACCTTAGTG TGACCGAAT; m-MuRF-1, sense: TTCCTCTCAAGTGCC AAG, anti-sense: GCCTCTGCTATGTGT TCTA; m-Beclin-1, sense: GGAGACTCAAGGTCACTG, anti-sense: TCACTGTCATCCTCATTCAT; m-TNF-α, sense: CACGTCGTAGCAAACCACCAAGTGGA, anti-sense, TGGGAGTAGACAAGGTACAACCC; m-IL-1β, sense: TCAACCAACAAGTGATATTCTC, anti-sense: TTACACAGCACAGGTATAGATT; m-18s, sense: GAAGTACAGCCAGGTTCT, anti-sense ACTCATTTCTTCTTGGATACAC. Relative gene expression levels were calculated using the 2-ΔΔCt method, normalized to the internal control 18S.

### 2.7. Glylcogen, Proinflammatory Cytokine, and Lipid Peroxidation Levels

The levels of glycogen in the triceps muscle tissue were determined using a Glycogen Colorimetric/Fluorometric Assay kit (BioVision, Atlanta, GA, USA). We determined the glycogen of the triceps muscle because this muscle is the muscle located in the forelimb, which was used to measure the MGS [21].

The proinflammatory cytokine, TNF-α and IL-6, concentration in the plasma was determined using an enzyme-linked immunosorbent assay (ELISA) kit (Thermo Fisher, Waltham, MA, USA) as described by the manufacturer.

The diluted red blood cells and homogenized GA were used to assess thiobarbituric acid reactive substances (TBARs) levels. Samples were added to a reaction mixture containing 15% (*w*/*v*) TCA, 0.38% (*w*/*v*) TBA, and 1.6% (*w*/*v*) antioxidant BHT, then mixed thoroughly. The mixtures were incubated at 100 °C for 20 min. After cooling, samples were centrifuged at 4000× *g* for 10 min. The concentrations of TBARs in the supernatant were determined at 532 nm using the SpectraMax M5 Microplate Reader (Molecular Devices, LLC, San Jose, CA, USA). The absorbance values were then used to determine the TBARs content in each sample based on a malondialdehyde standard curve prepared using 1,1,3,3-tetramethoxypropane.

### 2.8. Statistical Analysis

Data are expressed as mean ± SD and analyzed for statistical significance using Student’s *t*-test for two group comparisons or one-way analysis of variance (ANOVA) followed by Duncan’s multiple range test for group mean comparisons. Statistical analysis was performed using the Statistical Analysis System (SAS, Cary, NC, USA). A *p*-value of less than 0.05 was considered statistically significant.

## 3. Results

### 3.1. PE Reduces Body Weight Loss and Increases Food Intake in Tumor-Bearing Mice

Before tumor cell injection, there was no significant difference in body weight between the groups (Table 1). Three weeks after tumor cell injection, the net body weight (body weight without the tumor) of the tumor-only group was significantly lower than that of the control group (*p* < 0.001). After tumor cell injection, the food and water intake of the tumor-only group significantly decreased compared to the control group (*p* < 0.001). Although there was no significant difference among the tumor-bearing groups, PE but not tryptone supplementation promoted the recovery of net body weight and food intake at weeks 1 and 3 without affecting tumor weight when compared to the tumor-only group (*p* < 0.05, Student’s *t*-test).

### 3.2. PE Prevents MLGS Reduction, Inflammation, Oxidative Stress, and Muscle Atrophy in Tumor-Bearing Mice

Compared to the control group, MLGS and muscle weights (triceps, gastrocnemius, and quadriceps), measured three weeks after tumor cell injection and at the time of sacrifice, respectively, were significantly reduced in the tumor-only group (Table 2, *p* < 0.001). PE supplementation significantly restored the decrease in MLGS (*p* < 0.05). Although there was no significant difference among the tumor-bearing groups, PE supplementation but not tryptone markedly increased muscle weights compared to the tumor-only group (*p* < 0.05, Student’s *t*-test).

The tumor-only group also significantly increased IL-6 and TBAR levels in the plasma, as well as the IL-1β mRNA level in the gastrocnemius, compared to the control group (Table 2, *p* < 0.001). PE supplementation significantly suppressed the increase in all these proinflammatory cytokines and oxidative stress markers induced by tumor-bearing. Tryptone supplementation also decreased the levels of TBARs and IL-6 in the plasma (Table 2, *p* < 0.05).

**Figure 1:**
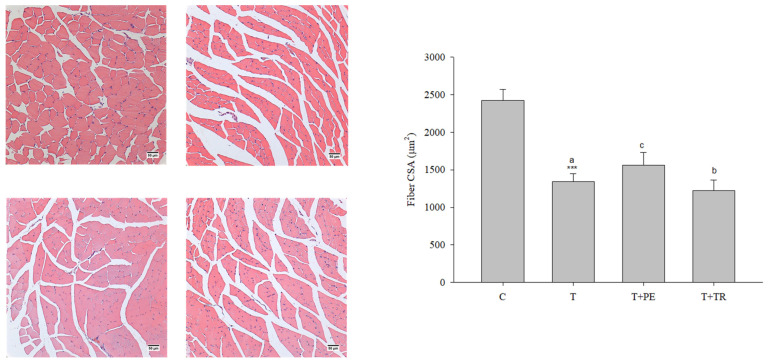
The effects of perch essence (PE) and tryptone (TR) supplementation on H&E-stained images (scale bar: 50 μm) and the cross-sectional area (CSA) of muscle fibers in the quadriceps muscle of mice injected with tumor cells (T) were analyzed. Values are expressed as means ± SD. Statistical significance is indicated by *** (*p* < 0.001) for comparisons between the tumor-only (T) group and the control group (Student’s *t*-test). Tumor cell-treated groups that do not share a common letter are significantly different (*p* < 0.05, ANOVA).

**Figure 2:**
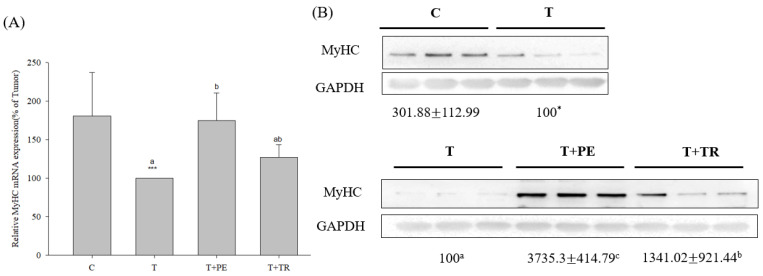
The effects of perch essence (PE) and tryptone (TR) supplementation on MyHC mRNA (**A**) and protein expression (**B**) in the gastrocnemius muscle of mice injected with tumor cells. Values are expressed as means ± SD (*n* = 8~10). Statistical significance is indicated by * (*p* < 0.05) and *** (*p* < 0.001) for comparisons between the tumor-only (T) group and the control group (Student’s *t*-test). Tumor cell-treated groups that do not share a common letter are significantly different (*p* < 0.05, ANOVA).

Furthermore, we measured fiber size in the quadriceps muscles, as well as the mRNA and protein levels of MyHC, an indicator of muscle protein content, in the gastrocnemius muscle. The results consistently showed that the muscle fiber CSAs, along with MyHC mRNA and protein levels, were significantly decreased in the tumor-only group compared to the control group (Figure 1 and Figure 2, respectively). In contrast, PE supplementation significantly restored these parameters in tumor-bearing mice (*p* < 0.05). Tryptone supplementation also increased MyHC expression, but its efficiency was lower than that of PE.

### 3.3. PE Ameliorates the Expression of Muscle-Degraded and Apoptosis-Associated Proteins

**Figure 3:**
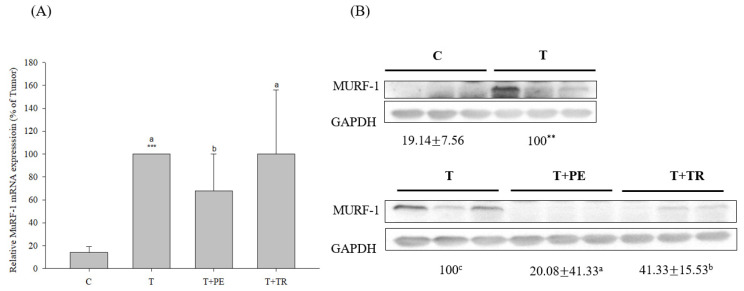
The effects of perch essence (PE) and tryptone (TR) supplementation on MuRF-1 mRNA (**A**) and protein expression (**B**) in the gastrocnemius muscle of mice injected with tumor cells. Values are expressed as means ± SD (*n* = 8~10). Statistical significance is indicated by ** (*p* < 0.01) and *** (*p* < 0.001) for comparisons between the tumor-only (T) group and the control group (Student’s *t*-test). Tumor cell-treated groups that do not share a common letter are significantly different (*p* < 0.05, ANOVA).

**Figure 4:**
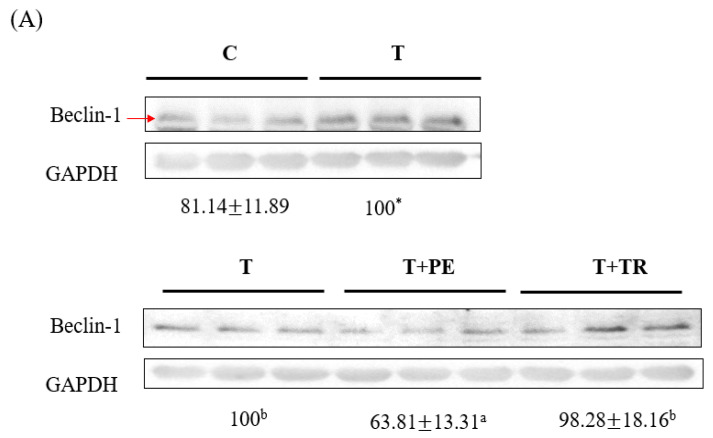

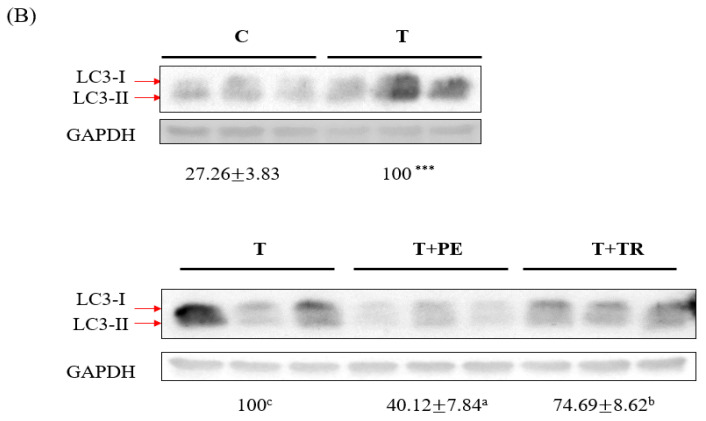
The effects of perch essence (PE) and tryptone (TR) supplementation on Beclin-1 (**A**) and LC3-II (**B**) protein expression in the gastrocnemius muscle of mice injected with tumor cells. Values are expressed as means ± SD (*n* = 8~10). Statistical significance is indicated by * (*p* < 0.05) and *** (*p* < 0.001) for comparisons between the tumor-only (T) group and the control group (Student’s *t*-test). Tumor cell-treated groups that do not share a common letter are significantly different (*p* < 0.05, ANOVA).

To investigate the mechanisms by which PE supplementation exerts its protective effect on muscle atrophy, we measured the expression levels of MURF-1, a key E3 ubiq-uitin ligase in the UPP, as well as Beclin-1 and LC3-II, markers of the ALP. Both pathways are associated with muscle atrophy [7,8,29]. As shown in Figure 3 and Figure 4, the gastrocnemius muscles in the tumor-only group exhibited significantly in-creased expression of MURF-1, Beclin-1, and LC3-II compared to the control group (*p* < 0.001). PE supplementation significantly decreased the expression of MURF-1, Beclin-1, and LC3-II induced by tumor cell injection (*p* < 0.05). Tryptone supplementation also reduced their expression but was less effective than PE.

### 3.4. PE Supplementation Increases p-Akt, p-FoxO1 but Decreases p-NF-κB p65 Protein Expression in the Gastrocnemius Muscle in Cancer-Bearing Mice

**Figure 5:**
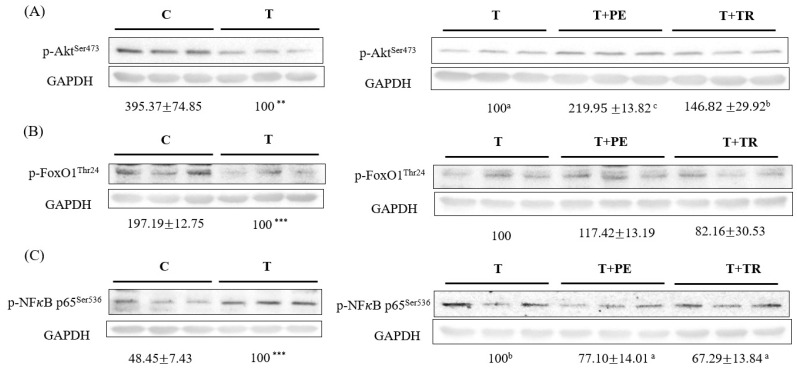
The effects of perch essence (PE) and tryptone (TR) supplementation on the protein expression of p-Akt (**A**), p-FoxO1 (**B**), and p-NFκB p65 (**C**) in the gastrocnemius muscle of mice injected with tumor cells. Values are expressed as means ± SD (*n* = 8~10). Statistical significance is indicated by ** (*p* < 0.01) and *** (*p* < 0.001) for comparisons between the tumor-only (T) group and the control group (Student’s *t*-test). Tumor cell-treated groups that do not share a common letter are significantly different (*p* < 0.05, ANOVA).

It is well established that the activation of NF-κB and FOXO is crucial for UPP- and ALP-related gene expression [6,7,15,17]. Moreover, Akt activation inhibits the expression of muscle atrophy-related genes through diminishing FOXO activation [18]. As shown in Figure 5, compared to the control group, the tumor significantly decreased phosphorylated Akt and FOXO1 levels while increasing phosphorylated NF-κB p65 expression, which represents the activation of FOXOs and NF-κB, respectively, in the gastrocnemius muscle (*p* < 0.01 or 0.001). PE supplementation prominently restored the levels of p-Akt, p-FoxO1, and p-NF-κB p65 altered by the tumor. The protective potency of tryptone was lower than that of PE.

### 3.5. PE Increases Antioxidant Enzymes in Gastrocnemius Muscle in Tumor-Bearing Mice

**Figure 6:**
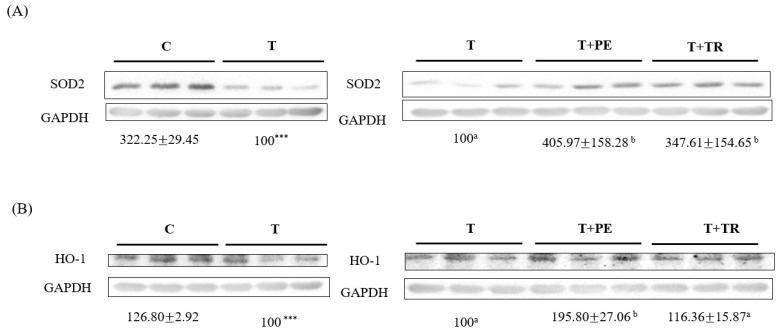
The effects of perch essence (PE) and tryptone (TR) supplementation on the protein expression of antioxidant enzymes SOD2 (**A**) and HO-1 (**B**) in the gastrocnemius muscle of mice injected with tumor cells. Values are expressed as means ± SD (*n* = 8~10). Statistical significance is indicated by *** (*p* < 0.001) for comparisons between the tumor-only (T) group and the control group (Student’s *t*-test). Tumor cell-treated groups that do not share a common letter are significantly different (*p* < 0.05, ANOVA).

We further determined the expression of antioxidant enzymes in the gastrocnemius muscle. The results showed that compared to the control group, the SOD-2 and HO-1 protein levels in the gastrocnemius muscle of the tumor-only group significantly decreased (*p* < 0.001; Figure 6). PE supplementation, which was more potent than tryptone, significantly increased SOD2 and HO-1 protein expression in the gastrocnemius muscle of tumor-bearing mice (*p* < 0.05).

### 3.6. PE Suppresses Cisplatin-Induced Muscle Atrophy

Except for cancer cachexia, we also used BALB/c mice to assess the effect of PE supplementation on muscle atrophy induced by CDDP, a common chemotherapy agent. Compared to the control group, CDDP administration significantly reduced food intake starting from week 2 (Table 3). Food intake recovered approximately 4 days later, but the recovery was attenuated after 6 weeks of CDDP treatment. Compared to the CDDP-alone group, 2XPE showed a trend toward restoring food intake, particularly at weeks 3, 5, and 7 after CDDP administration (*p* < 0.05). The effects of 1XPE and tryptone were generally not significant. At the end of the study, body weights and muscle weights (triceps, quadriceps, and gastrocnemius) in the CDDP-alone group were significantly lower than those in the control group (Table 4). Both 2XPE and 1XPE, but not tryptone supplementation, significantly recovered gastrocnemius weight loss induced by CDDP (*p* < 0.05).

In addition, CDDP alone also significantly reduced MLGS (by approximately 30–37%) and the muscle glycogen level but significantly increased the mRNA level of proinflammatory cytokines, TNF-α and IL-1β, compared to the control group (*p* < 0.05). Both 2XPE and 1XPE significantly recovered changes in MLGS, muscle glycogen levels, and proinflammatory cytokine concentrations in the plasma and mRNA levels in the gastrocnemius in mice exposed to CDDP (*p* < 0.05). Tryptone significantly improved CDDP-induced changes in MLGS and some proinflammatory cytokine parameters, but had no effect on muscle glycogen level and IL-1β mRNA level.

**Figure 7:**
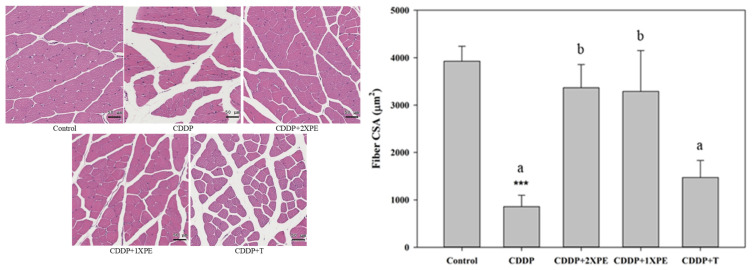
The effects of perch essence (1XPE and 2XPE, 0.024 g/d and 0.048 g/d, respectively) and tryptone (TR) on H&E staining images with scale bar 50 μm (**A**) and mean cross-sectional area (CSA) of muscle fiber (**B**) in the quadriceps muscle of BALB/c mice exposed to cisplatin (CDDP). Values are expressed as means ± SD. Values are expressed as means ± SD. Statistical significance is indicated by *** (*p* < 0.001) for comparisons between the CDDP group and the control group (Student’s *t*-test). CDDP-treated groups that do not share a common letter are significantly different (*p* < 0.05, ANOVA).

**Figure 8:**
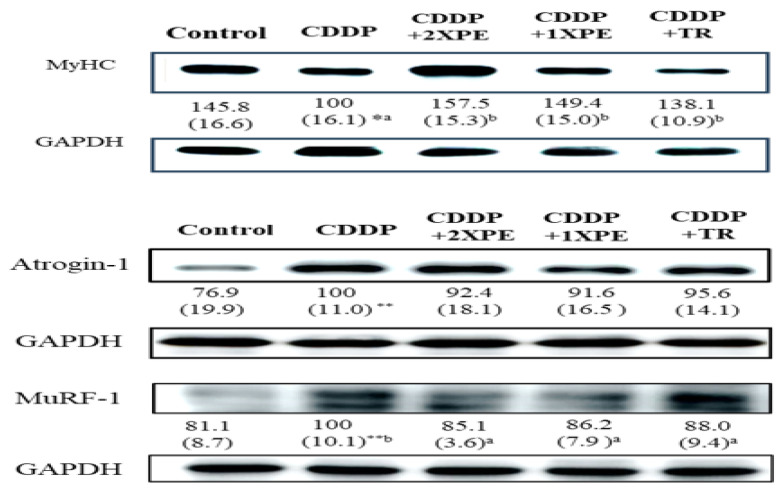
The effects of perch extract (1XPE and 2XPE, 0.024 g/d and 0.048 g/d, respectively) and tryptone (TR) on protein expression of MyHC, Atrogin-1, and MuRF1 in the gastrocnemius muscle of BALB/c mice treated with cisplatin (CDDP). Values are expressed as means ± SD (*n* = 6~8). Statistical significance is indicated by * (*p*< 0.05), ** (*p* < 0.01) for comparisons between the CDDP group and the control group (Student’s *t*-test). CDDP-treated groups that do not share a common letter are significantly different (*p* < 0.05, ANOVA).

Furthermore, CDDP alone significantly decreased the cross-sectional area of muscle fibers and the MyHC protein expression compared to the control group (Figure 7 and Figure 8, respectively), while significantly increased the protein expression of Atrogin-1 and MuRF1 (Figure 8). The supplementation with two doses of PE demonstrated a significant and similar recovery in muscle fiber CSA and MyHC expression but reduced Atrogin-1 and MuRF1 expression in mice exposed to CDDP (*p* < 0.05). Tryptone supple mentation exhibited a less or similar recovery effect on these parameters compared to 2XPE.

## 4. Discussion

Meat extract or essence soup, produced under high-temperature, high-pressure conditions to break down protein macromolecules into smaller peptides, is a popular worldwide nutraceutical supplement with demonstrated health and disease-alleviating benefits [30]. The PE used in this study, derived from perch head, bones, meat, and skin, exhibited therapeutic potential for metabolic syndrome and muscle health [23,24,27]. The present data demonstrate, for the first time, that PE supplementation not only increases food intake but also prevents body weight loss as well as skeletal muscle atrophy and dysfunction in C26 tumor-bearing mice. Notably, the remedial effects of PE supplementation on cancer cachexia are more potent than equivalent protein supplementation from tryptone (a casein hydrolysate), suggesting that factors beyond increased protein and BCAA intake contribute to PE supplementation’s therapeutic value. In addition to being lipid-free and rich in BCAAs, the PE used in this study contains 32.4% small peptides [25]. Although there are no data showing that marine-derived small peptides can counteract cancer cachexia-induced muscle atrophy, these peptides exhibit more than 45 different biological activities, including antioxidant, anti-inflammatory, and antifatigue, and anticancer effects, which may contribute to protecting against muscle atrophy caused by cancer cachexia [10,11,31,32,33,34]. More studies are needed to identify the specific components of PE that offer greater therapeutic value than the total protein content in treating cancer cachexia.

To investigate the mechanisms underlying the therapeutic benefits of PE supplementation on cancer cachexia-induced muscle atrophy, we assessed the effects of PE on the UPP and ALP, both related to protein catabolism, as well as on inflammation and oxidative stress, known triggers of protein catabolism [6,7,10,11]. Our data indicate an increased expression of the muscle-specific E3 ubiquitin ligase involved in the UPP, as well as elevated levels of the autophagy-initiating protein beclin-1 and the autophagosome marker protein LC3-II in the gastrocnemius muscles of C26 tumor-bearing mice. Moreover, cancer cachexia not only elevated systemic inflammatory cytokine levels and lipid peroxidation but also increased inflammatory cytokine mRNA expression and reduced antioxidant enzyme expression in the gastrocnemius muscles of mice. Supplementation with PE was more effective than tryptone in counteracting all cancer cachexia-induced changes. Interestingly, PE supplementation also modulated the phosphorylation levels of AKT, FOXO, and NF-κB p65, key regulators involved in the induction of atrogene expression associated with the UPP and ALP [6,7,15,17]. Given that elevated UPP and ALP markers, along with increased inflammation and oxidative stress, are commonly observed in various clinical settings of muscle atrophy [9,10], it is worthwhile to investigate the therapeutic potential of PE supplementation in muscle atrophy induced by other chronic or acute diseases.

Furthermore, the present study showed that PE supplementation also suppressed cisplatin-induced loss of body weight, muscle weight, and MLGS. In agreement with the findings observed in tumor-bearing mice, the general protective effects of PE supplementation on CDDP-induced adverse changes were more potent than the equivalent amount of protein from tryptone. This finding supports the speculation mentioned above that the factors present in PE beyond increased protein intake contribute to the protective effects of PE. A study by Song et al. [35] showed that fish skin collagen-derived peptides, weighing approximately 0.4 to 1 kDa, attenuate cytotoxicity and oxidative damage through mechanisms associated with inhibiting the mitogen-activated protein kinase (MAPK) signaling pathway in mouse thymic epithelial cells. Although we did not identify the peptides present in PE, a study by Lin et al. [36] demonstrated that enzyme-digested peptides, with molecular weights ranging from 1 to 5 kDa, from this species of fish (*Lates calcarifer*), enhanced microvessel formation and accelerated the wound healing process in mice. However, further studies are needed to investigate the precise compounds contributing to the effects observed in the present study.

Studies including our previous study have demonstrated that CDDP causes loss of MLGS by inducing muscle atrophy through the activation of the Akt/FoxO1/MuRF1/Atrogin-1 signaling pathway and increasing proinflammatory cytokines [22]. The present study showed that PE also suppressed CDDP-induced muscle atrophy by decreasing protein degradation, as evidenced by the recovery of the CSA and MyHC levels. The mechanisms were also associated with the downregulation of the UPP and proinflammatory cytokine levels, as observed in tumor-bearing mice.

Besides the prevention of the loss of muscle mass, the protective effect of PE on CDDP-induced loss of MLGS was also associated with the increase in glycogen in the muscle of mice exposed to CDDP. CDDP has been shown to reduce muscle strength and activity partly through decreasing glycogen storage levels in skeletal muscle in mice [21,37]; in contrast, PE restored the loss. Our findings were in agreement with those of Chen et al. [23], who demonstrated that PE supplementation extended swimming endurance time in rats by increasing glycogen concentration in the gastrocnemius muscle and liver.

Skeletal muscle, comprising 30 to 40% of body weight, is essential for posture, locomotion, breathing, macromolecule metabolism, and oxygen consumption. Additionally, it contains 50–75% of the body’s protein, serving as a significant reservoir [38]. Cachexia, induced by tumors, chemotherapy, and other chronic diseases, accelerates skeletal muscle protein breakdown and amino acid release over protein synthesis, resulting in the loss of muscle mass and strength [3,5,11]. Beyond being a prominent phenotypic feature of cancer cachexia, muscle atrophy not only devastates patients’ quality of life but also reduces the effectiveness of disease treatments, ultimately contributing to patient mortality [39]. To achieve fewer side effects and more promising outcomes, various studies explore the use of functional dietary supplements instead of synthetic drugs to prevent and reduce cachexia-induced muscle atrophy. The present study demonstrated that beyond the amounts of protein and BCAA, PE supplementation containing low molecular weight peptides was more effective in improving muscle atrophy and dysfunction induced by cancer cachexia and CDDP. This effect was achieved by reducing inflammation and oxidative stress, as well as inhibiting NF-kB and FOXO activation, thereby diminishing the UPP and ALP induced by cachexia. Based on our preclinical data, it is worthwhile to conduct a human study to explore the protective effect of 1 to 3 packs of PE supplementation per day on muscle atrophy in patients with cancer cachexia and in those undergoing chemotherapy.

## Figures and Tables

**Table 1:** The effects of perch essence (PE) and tryptone (TR) supplementation on body weight, tumor weight, food intake, and water intake in mice injected with tumor cells (T).

	C	T	T+PE	T+TR
Body weight				
Before tumor cell injection (g)	19.64 ± 1.29	20.42 ± 1.16	20.08 ± 0.90	20.70 ± 1.34
Net body weight three weeks after tumor cell injection (g)	25.50 ± 0.97	17.39 ± 1.72 *	19.22 ± 1.19	18.66 ± 1.58
Change in body weight (%)	28.74 ± 5.50	−13.45 ± 6.15 ***	−7.38 ± 6.17	−9.92 ± 5.59
Tumor weight	-	1958.02 ± 261.92	1932.15 ± 170.69	2006.81 ± 210.23
Food intake				
Week 1	4.11 ± 0.35	3.38 ± 0.18 ***	3.28 ± 0.07	3.37 ± 0.20
Week 2	4.19 ± 0.37	2.82 ± 0.37 ***	2.88 ± 0.18	2.82 ± 0.48
Week 3	3.84 ± 0.43	1.78 ± 032 ***	2.06 ± 0.45	1.95 ± 0.32
Water intake				
Week 1	5.02 ± 0.50	4.40 ± 0.32 ***	4.65 ± 0.49	4.56 ± 0.46
Week 2	5.42 ± 0.53	4.56 ± 0.52 ***^a^	5.04 ± 0.62 ^b^	4.93 ± 0.83 ^a^
Week 3	5.26 ± 1.18	3.92 ± 1.22 ***	4.03 ± 0.62	3.87 ± 0.90

Values are expressed as means ± SD. Statistical significance is indicated by * (*p* < 0.05), *** (*p* < 0.001) for comparisons between the tumor-only (T) group and the control group (Student’s *t*-test). Tumor cell-treated groups that do not share a common letter are significantly different (*p* < 0.05, ANOVA).

**Table 2:** The effects of perch essence (PE) and tryptone (TR) supplementation on maximum limb grip strength (MLGS), muscle weight, plasma IL-6 concentration, gastrocnemius IL-1β mRNA expression, and red blood cells TBARs in mice injected with tumor cells (T).

	C	T	T+PE	T+TR
MLGS				
Before tumor-bearing	109.96 ± 3.44	107.72 ± 5.73	108.40 ± 9.30	108.78 ± 5.16
Three weeks after tumor-bearing	167.71 ± 6.66	121.16 ± 18.59 ***^a^	140.22 ± 11.70 ^b^	125.38 ± 12.72 ^a^
Muscle Weight	**C**	**T**	**T+PE**	**T+TR**
Triceps muscle (mg)	98.35 ± 11.28	64.21 ± 8.14 ***	71.26 ± 7.51	68.14 ± 7.99
Quadriceps muscle (mg)	164.45 ± 13.85	88.39 ± 11.63 ***	102.02 ± 17.74	97.66 ± 11.55
Gastrocnemius muscle (mg)	126.85 ± 7.40	78.39 ± 13.21 ***	87.80 ± 9.64	85.65 ± 10.85
Plasma IL-6 concentration (pg/μL)	1.83 ± 1.78	182.6 ± 48.03 ***^b^	134.3 ± 46.48 ^a^	119.52 ± 51.46 ^a^
Gastrocnemius IL-1β mRNA (%)	45 ± 15	100 ***^b^	57 ± 13 ^a^	92 ± 43 ^b^
Red blood cells TBARs (MDA μmole/g protein)	0.69 ± 0.03	0.75 ± 0.04 **^c^	0.70 ± 0.03 ^b^	0.62 ± 0.05 ^a^

Values are expressed as means ± SD. Statistical significance is indicated by ** (*p* < 0.01), *** (*p* < 0.001) for comparisons between the tumor-only (T) group and the control group (Student’s *t*-test). Tumor cell-treated groups that do not share a common letter are significantly different (*p* < 0.05, ANOVA).

**Table 3:** The effects of perch essence (1XPE and 2XPE, 0.024 g/d and 0.048 g/d, respectively) and tryptone (TR) on the food intake in BALB/c mice exposed to cisplatin (CDDP).

Food Intake (g/day)						
Week	Day	Control	CDDP	CDDP+2XPE	CDDP+1XPE	CDDP+TR
**1**	4 ^	3.6 ± 0.4	2.4 ± 0.6	2.8 ± 0.1	2.6 ± 0.3	3.1 ± 0.2
	8	3.4 ± 0.1	3.5 ± 0.3	3.4 ± 0.2	3.3 ± 0.2	3.5 ± 0.1
**2**	11 ^	3.7 ± 0.1	2.7 ± 0.2 *	3.0 ± 0.2	3.0 ± 0.2	2.9 ± 0.4
	15	3.6 ± 0.1	3.1 ± 0.3	3.3 ± 0.1	3.0 ± 0.3	3.2 ± 0.2
**3**	18 ^	3.5 ± 0.0	2.5 ± 0.2 **^a^	3.1 ± 0.4 ^b^	2.6 ± 0.2 ^ab^	2.7 ± 0.3 ^ab^
	22	3.6 ± 0.1	3.1 ± 0.1 *	3.4 ± 0.1	3.0 ± 0.3	3.2 ± 0.2
**4**	25 ^	3.4 ± 0.5	2.1 ± 0.1	2.6 ± 0.5	2.4 ± 0.2	2.4 ± 0.3
	29	3.5 ± 0.1	3.0 ± 0.1 *	3.4 ± 0.2	3.0 ± 0.4	3.1 ± 0.4
**5**	32 ^	3.8 ± 0.1	1.9 ± 0.1 ***^a^	2.6 ± 0.1 ^b^	2.2 ± 0.4 ^ab^	2.3 ± 0.1 ^ab^
	36	3.7 ± 0.1	2.6 ± 0.2 **	3.2 ± 0.2	2.7 ± 0.4	3.0 ± 0.5
**6**	39 ^	4.1 ± 0.0	2.5 ± 0.3 **	2.7 ± 0.2	2.5 ± 0.3	2.7 ± 0.4
	43	4.0 ± 0.3	2.8 ± 0.1 **	3.3 ± 0.2	2.9 ± 0.4	3.1 ± 0.4
**7**	46 ^	3.7 ± 0.4	2.0 ± 0.2 ^a^	2.5 ± 0.1 ^b^	2.4 ± 0.4 ^ab^	2.6 ± 0.4 ^ab^
	50	3.9 ± 0.2	2.4 ± 0.2 **^a^	3.6 ± 0.3 ^b^	2.7 ± 0.4 ^a^	3.1 ± 0.6 ^ab^
**8**	53 ^	4.0 ± 0.1	2.1 ± 0.3 **	2.6 ± 0.4	2.3 ± 0.3	2.4 ± 0.5
	57	3.8 ± 0.2	2.1 ± 0.1 ^a^	3.0 ± 0.2 ^b^	2.5 ± 0.5 ^ab^	2.7 ± 0.6 ^ab^
**9**	60 ^	3.4 ± 0.2	1.6 ± 0.3 **	2.9 ± 0.7	2.6 ± 0.3	2.8 ± 1.0

Values are expressed as means ± SD. Statistical significance is indicated by * (*p*< 0.05), ** (*p* < 0.01), *** (*p* < 0.001) for comparisons between the CDDP group and the control group (Student’s *t*-test). CDDP-treated groups that do not share a common letter are significantly different (*p* < 0.05, ANOVA). The symbol ^ indicates the day of CDDP injection.

**Table 4:** The effects of perch essence (1XPE and 2XPE, 0.024 g/d and 0.048 g/d, respectively) and tryptone (TR) on body weight, muscle weight, maximum limb grip strength (MLGS), muscle glycogen, plasma and gastrocnemius proinflammatory cytokine levels, and gastrocnemius TBARs in BALB/c mice exposed to cisplatin (CDDP).

	Control	CDDP	CDDP +2XPE	CDDP +1XPE	CDDP+TR
Body weight					
Before CDDP injection (g)	20.6 ± 2.0	20.5 ± 1.2	20.6 ± 1.2	20.5 ± 0.8	20.7 ± 1.5
At the end of the experiment (g)	28.7 ± 2.1	19.6 ± 2.0 ***^a^	23.6 ± 2.5 ^b^	22.4 ± 3.1 ^b^	22.8 ± 1.9 ^b^
Change at the end of the experiment (g)	8.4 ± 1.3	−0.9 ± 2.3 ***^a^	3.0 ± 1.8 ^b^	1.8 ± 3.0 ^b^	2.1 ± 2.6 ^b^
Muscle weight					
Triceps muscle (mg)	200.3 ± 22.7	162.9 ± 20.3 **	187.2 ± 30.7	169.6 ± 16.5	169.7 ± 20.1
Quadriceps muscle (mg)	417.8 ± 68.1	332.3 ± 44.2 **	361.9 ± 48.0	352.4 ± 50.3	352.0 ± 26.9
Gastrocnemius muscle (mg)	294.1 ± 21.9	230.4 ± 14.1 ***^a^	264.6 ± 17.5 ^b^	291.9 ± 12.8 ^c^	242.5 ± 14.8 ^a^
MLGS					
At week 4 (gf)	106.8 ± 7.9	73.5 ± 18.2 ***^a^	121.6 ± 16.9 ^c^	109.8 ± 12.1 ^bc^	104.2 ± 12.8 ^b^
At week 8 (gf)	116.1 ± 10.7	72.8 ± 14.4 ***^a^	111.5 ± 11.0 ^c^	104.4 ± 11.1 ^c^	88.6 ± 14.0 ^b^
Triceps muscle glycogen (mg/g protein)	49.4 ± 3.8	38.6 ± 4.7 **^a^	47.1 ± 3.6 ^b^	46.1 ± 2.3 ^b^	35.0 ± 3.5 ^a^
Plasma TNF-α concentration (pg/mL)	5.8 ± 0.8	7.9 ± 1.3 **^c^	3.9 ± 1.2 ^b^	2.1 ± 0.4 ^a^	2.4 ± 0.9 ^a^
Plasma IL-6 concentration (pg/mL)	12.7 ± 0.6	16.3 ± 1.0 ***^c^	11.1 ± 1.0 ^b^	9.35 ± 1.0 ^a^	11.2 ± 0.7 ^b^
Gastrocnemius TNF-α mRNA (%)	53 ± 17	100 ± 0 *^b^	34 ± 21 ^a^	43 ± 16 ^a^	37 ± 23 ^a^
Gastrocnemius IL-1β mRNA (%)	29 ± 13	100 ± 0 ***^b^	40 ± 10 ^a^	40 ± 20 ^a^	141 ± 40 ^c^
Gastrocnemius TBARs (malondialdehyde mol/mg protein)	0.3 ± 0.0	0.3 ± 0.0 ^b^	0.3 ± 0.0 ^ab^	0.2 ± 0.0 ^a^	0.3 ± 0.0 ^b^

Values are expressed as means ± SD. Statistical significance is indicated by * (*p*< 0.05), ** (*p* < 0.01), *** (*p* < 0.001) for comparisons between the CDDP group and the control group (Student’s *t*-test). CDDP-treated groups that do not share a common letter are significantly different (*p* < 0.05, ANOVA).

## Data Availability

The data presented in this study are available on request from the corresponding author.
